# Supplementary material for: Content-rich biological network constructed by mining PubMed abstracts
Source: BMC Bioinformatics. 2004 Oct 8;5:147. doi: 10.1186/1471-2105-5-147 (PMC528731; doi:10.1186/1471-2105-5-147)
Supplement: Additional File 2 — The original results of the above study (non-essential files are deleted to keep the file size under the limit set by BMC bioinformatics). [file 1471-2105-5-147-S2.bz2 › chilibotAdditionalFile2/dip20/341ID8076595E4256/html/TNFSF4_TNFRSF4.html]

 


 **TNFSF4** and **TNFRSF4** 
  
Found 114 abstracts in PubMed, retrieved 20.  
 

 What does Google say? 
 PDF only 
| .edu only 

---

**Interactive relationship** (e.g. stimulation, inhibition, etc)

**Neutral relationship**- Thus, OX40  [ **TNFRSF4** ]  OX40L  [ **TNFSF4** ]  interactions are pivotal to the efficiency of recall responses regulated by memory Th2 cells.  Ref: 12860930 J Exp MedJ Exp Med,
- Interaction of OX40  [ **TNFRSF4** ]  CD134  [ **TNFRSF4** ]  and its ligand, OX40L  [ **TNFSF4** ] , has been implicated in T cell activation and cell migration.  Ref: 12794142 J Immunol, 2003
- In already sensitized animals, blocking OX40  [ **TNFRSF4** ]  OX40L  [ **TNFSF4** ]  interactions at the time of inhalation of aerosolized antigen suppressed memory effector accumulation in lung draining lymph nodes and lung, and prevented eosinophilia, airway hyperreactivity, mucus secretion, and Th2 cyto kine production.  Ref: 12860930 J Exp MedJ Exp Med, 2003
- The interaction between OX40  [ **TNFRSF4** ]  and OX40  [ **TNFRSF4** ]  ligand  [ **TNFSF4** ]  OX40L  [ **TNFSF4** ]  is suggested to provide T cells with an effective costimulatory signals during T cell APC interaction.  Ref: 12370402 J Immunol, 2002
- Constitutive OX40  [ **TNFRSF4** ]  OX40  [ **TNFRSF4** ]  ligand  [ **TNFSF4** ]  interaction induces autoimmune like diseases.  Ref: 12370402 J Immunol, 2002
- Blockade of OX40  [ **TNFRSF4** ]  OX40L  [ **TNFSF4** ]  interaction in the recipient RAG2 deficient mice completely prevented disease development.  Ref: 12370402 J Immunol, 2002
- The role of OX40  [ **TNFRSF4** ]  ligand  [ **TNFSF4** ]  interactions in the development of the Th2 response to the gastrointestinal nematode parasite Heligmosomoides polygyrus.  Ref: 12496423 J Immunol, 2003
- These studies suggest an important role for OX40  [ **TNFRSF4** ]  OX40L  [ **TNFSF4** ]  interactions in specifically promoting IL 4 production, as well as associated IgE elevations, in Th2 responses to H. polygyrus.  Ref: 12496423 J Immunol, 2003
- However, in vivo blockade of OX40  [ **TNFRSF4** ]  OX40L  [ **TNFSF4** ]  interactions reduced GVHD mortality induced by either signal transducer and activator of transcription 6 Stat 6 Th2 defective or Stat 4 Th1 defective major histocompatibility complex MHC disparate splenocytes, indicating that the GVHD ameliorating effects did not require Stat 4 or Stat 6 signaling.  Ref: 12521997 Blood, 2003
- CD40KO mice primed with C26 GM OX40L  [ **TNFSF4** ]  cells failed to mount a CTL response, and T cells infiltrating the C26 GM OX40L  [ **TNFSF4** ]  tumor were OX40  [ **TNFRSF4** ]  negative, suggesting an impairment in APC T cell cross talk in CD40KO mice.  Ref: 12496388 J Immunol, 2003
- Interaction of OX40  [ **TNFRSF4** ]  CD134  [ **TNFRSF4** ]  on T cells with its ligand OX40L  [ **TNFSF4** ]  on antigen presenting cells has been implicated in pathogenic T cell activation.  Ref: 12631559 Am J Physiol Gastrointest Liver Physiol, 2003
- To examine the in vivo effect of constitutive OX40  [ **TNFRSF4** ]  OX40L  [ **TNFSF4** ]  interaction during immune regulation, we report the establishment of OX40L  [ **TNFSF4** ]  transgenic OX40L  [ **TNFSF4** ]  Tg mice that constitutively express OX40L  [ **TNFSF4** ]  on T cells.  Ref: 12370402 J Immunol, 2002
- These results suggest that the OX40  [ **TNFRSF4** ]  OX40L  [ **TNFSF4** ]  system is crucial for induction of the allogeneic T cell response and the OX40  [ **TNFRSF4** ]  OX40L  [ **TNFSF4** ]  system is subsequent to and dependent on CD28 signalling, but is crucial for the end outcome of the human alloreactive T cell response.  Ref: 12757617 Immunology, 2003
- Coculture with MMCE gp34  [ **TNFSF4** ]  inhibited the apoptosis of Kit225 OX40  [ **TNFRSF4** ] , but Kit225 del OX40  [ **TNFRSF4** ]  apoptosis was hardly affected.  Ref: 12416737 Int J Hematol, 2002

**Non-interactive relationship** (e.g. studied together, co-existance, homology, etc.)

- This study was performed to explore the involvement of OX40  [ **TNFRSF4** ]  OX40L  [ **TNFSF4** ]  in the development of T cell mediated chronic colitis.  Ref: 12631559 Am J Physiol Gastrointest Liver Physiol, 2003
- OX40  [ **TNFRSF4** ]  ligand  [ **TNFSF4** ]  OX40L  [ **TNFSF4** ] , a member of TNF superfamily, is expressed on several APC such as splenic dendritic cells DC and activated B cells.  Ref: 12555678 Eur J Immunol, 2002
- In this study, we show that engagement of OX40  [ **TNFRSF4** ]  by an OX40L  [ **TNFSF4** ]  Ig fusion protein drives IFN gamma production by CD4 T cells and reduces eosinophilia and C. neoformans burden in the lung.  Ref: 12794142 J Immunol, 2003
- Our present results suggested a pivotal role of OX40  [ **TNFRSF4** ]  OX40L  [ **TNFSF4** ]  in the pathogenesis of T cell mediated chronic colitis.  Ref: 12631559 Am J Physiol Gastrointest Liver Physiol, 2003
- However, B cell costimulation via the OX40L  [ **TNFSF4** ]  OX40  [ **TNFRSF4** ]  pathway that has been implicated in CD4 cell expansion, survival, and Th2 development was required.  Ref: 12668647 J Exp Med, 2003
- Consequences of OX40  [ **TNFRSF4** ]  OX40  [ **TNFRSF4** ]  ligand  [ **TNFSF4** ]  interactions in langerhans cell function enhanced contact hypersensitivity responses in OX40L  [ **TNFSF4** ]  transgenic mice.  Ref: 12555678 Eur J Immunol, 2002
- Furthermore, in OX40L  [ **TNFSF4** ]  Tg mice administrated with superantigen, this constitutive OX40  [ **TNFRSF4** ]  OX40L  [ **TNFSF4** ]  interaction on CD4 T cells completely prevented normal in vivo clonal T cell deletion.  Ref: 12370402 J Immunol, 2002
- The CD134  [ **TNFRSF4** ]  CD134  [ **TNFRSF4** ]  ligand  [ **TNFSF4** ]  CD134L  [ **TNFSF4** ]  costimulatory pathway has been shown to be critical for both T and B cell activation.  Ref: 12626546 J Immunol, 2003
- its ligand, OX40  [ **TNFRSF4** ]  ligand  [ **TNFSF4** ]  OX40L  [ **TNFSF4** ]  is expressed on dendritic cells, B cells, and activated endothelial cells.  Ref: 12521997 Blood, 2003
- In these studies, we examined the effects of OX40  [ **TNFRSF4** ]  ligand  [ **TNFSF4** ]  OX40L  [ **TNFSF4** ]  deficiency on the development of Th2 cells during the Th2 immune response to the intestinal nematode parasite Heligmosomoides polygyrus.  Ref: 12496423 J Immunol, 2003
- We reported previously that OX40  [ **TNFRSF4** ] , a member of the tumor necrosis factor  [ **TNFSF4** ]  receptor family, is expressed constitutively on fresh leukemia lymphoma cells isolated from patients with adult T cell leukemia ATL.  Ref: 12416737 Int J Hematol, 2002
- Our data suggest that CD134  [ **TNFRSF4** ]  CD134L  [ **TNFSF4** ]  is a critical pathway in alloimmune responses, especially recall primed responses, and is synergistic with CD28 B7 in mediating T cell effector responses during allograft rejection.  Ref: 12626546 J Immunol, 2003
- OX40  [ **TNFRSF4** ]  ligand  [ **TNFSF4** ]  transduced tumor cell vaccine synergizes with GM CSF and requires CD40 Apc signaling to boost the host T cell antitumor response.  Ref: 12496388 J Immunol, 2003
- To determine how OX40  [ **TNFRSF4** ]  OX40L  [ **TNFSF4** ]  interaction affects graft versus host disease GVHD, we used antagonistic anti OX40L  [ **TNFSF4** ]  monoclonal antibody mAb or OX40  [ **TNFRSF4** ]  donor or OX40L  [ **TNFSF4** ]  recipient mice.  Ref: 12521997 Blood, 2003
- The costimulatory molecule OX40  [ **TNFRSF4** ]  ligand  [ **TNFSF4** ]  OX40L  [ **TNFSF4** ] , a member of the tumor necrosis factor  [ **TNFSF4** ]  superfamily, has been shown to be important in T cell priming and cytokine production.  Ref: 12355440 Eur J Immunol, 2002
- In the present study, we report the expression of both OX40L  [ **TNFSF4** ]  and OX40  [ **TNFRSF4** ]  on differentiated LC derived from draining lymph nodes in the FITC sensitized mice.  Ref: 12555678 Eur J Immunol, 2002
- To determine whether a cellular vaccine that interacts directly with both APC and T cells in vivo might be superior, we generated C26 carcinoma cells transduced with the T cell costimulatory molecule OX40  [ **TNFRSF4** ]  ligand  [ **TNFSF4** ]  OX40L  [ **TNFSF4** ]  either alone C26 OX40L  [ **TNFSF4** ]  or together with GM CSF C26 GM OX40L  [ **TNFSF4** ] , which is known to activate APC.  Ref: 12496388 J Immunol, 2003
- We first demonstrated that anti OX40  [ **TNFRSF4** ]  ligand  [ **TNFSF4** ]  anti OX40L  [ **TNFSF4** ]  monoclonal antibody mAb could markedly suppress the mixed leucocyte reaction MLR of peripheral blood mononuclear cells PBMC.  Ref: 12757617 Immunology, 2003
- The present results orchestrated in this study indicate that OX40  [ **TNFRSF4** ]  OX40L  [ **TNFSF4** ]  interaction may be a vital link in our understanding of T cell mediated organ specific autoimmunity.  Ref: 12370402 J Immunol, 2002
- Unlike dendritic cells, the CD4 CD3 cells have little CD80 or CD86 expression but do express high levels of the TNF ligands, OX40  [ **TNFRSF4** ]  ligand  [ **TNFSF4** ]  and CD30 ligand.  Ref: 12753741 Immunity, 2003
- We recently demonstrated a critical contribution of OX40  [ **TNFRSF4** ]  ligand  [ **TNFSF4** ]  OX40L  [ **TNFSF4** ]  to the development of Th2 mediated experimental leishmaniasis.  Ref: 12672051 Eur J Immunol, 2003
- We investigated the effect of CD134  [ **TNFRSF4** ]  CD134L  [ **TNFSF4** ]  pathway blockade on allograft rejection in fully MHC mismatched rat cardiac and skin transplantation models.  Ref: 12626546 J Immunol, 2003
- Crucially, the up regulation of OX40L  [ **TNFSF4** ]  on antigen presenting cells and CXCR 5 on T cells are CD40 dependent events and we show that T cells do not migrate to follicles in immunized OX40  [ **TNFRSF4** ]  deficient mice.  Ref: 12538659 J Exp Med, 2003
- These data provide insights as to the mechanisms responsible for OX40  [ **TNFRSF4** ]  OX40L  [ **TNFSF4** ]  regulation of GVHD.  Ref: 12521997 Blood, 2003
- To further define the contribution of the OX40  [ **TNFRSF4** ]  OX40L  [ **TNFSF4** ]  system to the MLR, we set up a co culture system of CD4 T cells and allogeneic monocyte derived dendritic cells DCs.  Ref: 12757617 Immunology, 2003
- Critical role for OX40  [ **TNFRSF4** ]  ligand  [ **TNFSF4** ]  in the development of pathogenic Th2 cells in a murine model of asthma.  Ref: 12672051 Eur J Immunol, 2003
- GVHD inhibition by blockade of the OX40  [ **TNFRSF4** ]  OX40L  [ **TNFSF4** ]  pathway did not require CD28 signaling.  Ref: 12521997 Blood, 2003
- In all these patients, the coculture of ATL cells with MMCE OX40  [ **TNFRSF4** ]  ligand  [ **TNFSF4** ]  gp34  [ **TNFSF4** ] , a stable human gp34  [ **TNFSF4** ]  transfectant of a mouse epithelial cell line, resulted in a decrease in the percentage of apoptotic cells after treatment with anti Fas monoclonal antibody, compared to coculture with MMCE mock controls.  Ref: 12416737 Int J Hematol, 2002
- The Role of the CD134  [ **TNFRSF4** ]  CD134  [ **TNFRSF4** ]  Ligand  [ **TNFSF4** ]  Costimulatory Pathway in Alloimmune Responses In Vivo.  Ref: 12626546 J Immunol, 2003
